# Immunomodulatory Effect of Raspberry (*Rubus idaeus* L.) Fruit Extracts on Activated Macrophages and Dysfunctional Vascular Endothelial Cells

**DOI:** 10.3390/nu17203257

**Published:** 2025-10-16

**Authors:** Katarzyna Kowalska, Radosław Dembczyński, Anna Olejnik

**Affiliations:** Department of Biotechnology and Food Microbiology, Poznan University of Life Sciences, 48 Wojska Polskiego St., 60-627 Poznan, Poland; katarzyna.kowalska@up.poznan.pl (K.K.); radoslaw.dembczynski@up.poznan.pl (R.D.)

**Keywords:** raspberry, anthocyanins, polyphenols, HUVECs, RAW 264.7, inflammation, anti-inflammatory, cytokine, chemokine

## Abstract

**Background:** Growing evidence highlights the beneficial effects of flavonoids, including anthocyanins, as key components in reducing cardiovascular risk, and emphasizes that incorporating anthocyanin-rich fruits into the daily diet significantly impacts public health. **Methods:** The effect of bioactive polyphenols from raspberry fruit (RBF) on molecular pathways in inflammation was studied in activated RAW 264.7 macrophages and their protective potential against endothelial dysfunction was analyzed using TNF-α-induced human umbilical vein endothelial cells (HUVECs). **Results:** The results have shown that RBF extract, along with its anthocyanin and polyphenol fractions, has a significant anti-inflammatory effect in macrophage cell culture by inhibiting the LPS-induced expression of pro-inflammatory genes, including IL-6, IL-1β, TNF-α, and NF-κB. Moreover, RBF and both fractions have demonstrated a protective effect on endothelial function by decreasing the expression of several inflammation-related genes and adhesion molecules, such as IL-6, IL-1β, VCAM-1, ICAM-1, and SELE, in TNF-α-induced HUVECs. **Conclusions:** The consumption of RBF and/or polyphenol-rich extracts may help prevent the onset of early atherosclerosis. This is attributed to their ability to improve inflammation status and enhance vascular endothelial function. Given the strong anti-inflammatory properties of RBF, incorporating them into a daily diet could significantly reduce the risk of non-communicable diseases related to inflammation.

## 1. Introduction

Cardiovascular disease (CVD) accounts for nearly half of all non-communicable disease deaths worldwide. The causes of CVD are well-known and include hypertension, high cholesterol, and age. Recently, CVD has also been recognized as being linked to chronic inflammation [1]. Atherosclerosis, the primary cause of CVD, is a chronic inflammatory condition in which immune cells produce pro-inflammatory cytokines. Macrophages, which can be either tissue-resident or derived from monocytes, play a crucial role in both the progression and regression of atherosclerosis [1,2]. Vascular macrophages influence the course of atherosclerosis by interacting with all cell types in the vessel wall. Pro-inflammatory macrophages are typically found in plaques that are progressing and actively inflamed [1,2].

The healthy endothelial monolayer that lines the vascular lumen regulates cell adhesion and vascular tone and maintains vascular homeostasis through anticoagulant, antithrombotic, and anti-inflammatory activities [3]. However, during atherosclerosis, the homeostatic properties of the endothelium are disrupted. This disruption is primarily due to the release of inflammatory cytokines by tissue-resident macrophages, such as TNF-α and IL-1β. These cytokines trigger the rapid expression of extracellular adhesion molecules, including E- selectin, P-selectin, intercellular adhesion molecule 1 (ICAM-1), and vascular cell adhesion molecule 1 (VCAM-1) [4]. While the accumulation of macrophages and their interactions with other cells in the vessel wall directly contribute to the development of atherosclerosis, these same macrophages are also essential for the stabilization and regression of atherosclerotic plaques [5].

The standard treatment for atherosclerosis primarily involves using lipid-lowering medications to decrease high circulating cholesterol level and reduce artery inflammation. However, due to the side effects associated with these medications, as well as the significance of the disease, there is a pressing need for accessible, safe, and low-cost anti-inflammatory therapies that specifically address inflammation [6]. Clinical trials have demonstrated that targeting vascular macrophages and inflammation can be an effective approach for treating CVD [6,7,8]. The CANTOS (Canakinumab Anti-inflammatory Thrombosis Outcomes Study) demonstrated that an antibody designed to neutralize IL-1β can reduce the recurrence of cardiovascular events in individuals who have sustained an acute myocardial infarction at least 30 days before enrollment. CANTOS proved the effectiveness of targeting inflammation in atherosclerosis and validated the inflammasome pathway as a promising path for further anti-inflammatory interventions [7].

Research suggests that a balanced diet may help prevent more than half of all cardiovascular events. Diets rich in fruits are the third most significant factor in reducing global rates of non-communicable diseases [9]. Increasing evidence highlights the beneficial effects of flavonoids, including anthocyanins, as key components in lowering the risk of CVD. Incorporating anthocyanin-rich fruits into the daily diet can significantly impact public health [10]. The best sources of anthocyanins are various berries, including blueberries, blackberries, raspberries, and strawberries. For instance, regular consumption of red raspberries has been linked to a reduced risk of chronic diseases [11]. Daily intake of these berries may help prevent the early development of atherosclerosis by enhancing antioxidant status and serum lipid profiles [12], eliminating vascular endothelial dysfunction [13], and exhibiting anti-inflammatory effects [14,15].

This study aimed to evaluate the effectiveness of red raspberry fruit (RBF) extract, as well as its anthocyanin (RBF-ACN) and non-anthocyanin polyphenol (RBF-PP) fractions, in preventing and treating endothelial dysfunction in vitro. The impact of RBF bioactive polyphenols on the molecular pathways associated with inflammation was analyzed using activated RAW 264.7 macrophages. The protective potential against endothelial dysfunction was assessed using TNF-α-induced human umbilical vein endothelial cells (HUVECs). Several crucial pro-inflammatory and anti-inflammatory cytokines were analyzed in inflamed cell culture models following treatment with RBF polyphenols.

## 2. Materials and Methods

### 2.1. Reagents

Dulbecco’s Modified Eagle’s Medium (DMEM), lipopolysaccharides (LPS) from *E. coli* O127, and TNF-α were purchased from Merck Life Science (Poznań, Poland). Fetal bovine serum (FBS) was obtained from Gibco BRL (Grand Island, NY, USA). Total RNA was isolated using the Tri Reagent from Merck Life Science (Poland). Template cDNA synthesis was carried out using the Transcriptor High Fidelity cDNA Synthesis Kit from Roche Diagnostics (Warsaw, Poland). PCR was performed with SYBR^®^ Select Master Mix from Life Technologies (Carlsbad, CA, USA). IL-6 and MCP-1 cytokines were quantified using ELISA kits from R&D Systems, Inc. (Minneapolis, MN, USA). All other reagents were sourced from Merck Life Science unless noted otherwise.

### 2.2. Preparation of Raspberry Fruit Extract and Anthocyanin and Non-Anthocyanin Polyphenol Fractions

Mature fruits of raspberry (*Rubus idaeus* L.) Polka cultivar were sourced as frozen products from Bio Berry Poland (Warsaw, Poland) and subsequently subjected to freeze-drying as described previously [16]. Two-stage freeze-drying process using a freeze-dryer LMC-1 (Christ, Hagen, Germany) was carried out with a vacuum pressure of 0.1 mbar, at 20 °C for 23 h followed by a 3 h post-drying phase at 23 °C.

The freeze-dried fruit powder was suspended in a 0.75% acetic acid solution and subjected to a two-step extraction process. The extract was purified from sugars and organic acids using an ÄKTA Explorer 100 Air chromatography system (GE Healthcare, Chicago, IL, USA) equipped with an XK 26/20 glass column (GE Healthcare, Chicago, IL, USA). This column was packed with Amberlite XAD-7 HP macroporous adsorbent resin (DuPont, Wilmington, DE, USA). Three eluents—5% formic acid, methanol, and 0.1% formic acid—were applied for elution. The entire effluent from the elution stage, which showed absorbance at 280, 320, and 520 nm, was evaporated at 30 °C using a rotary evaporator (Laborota 4003 HB control, Heidolph, Schwabach, Germany). The resulting solids were then dissolved in 0.75% acetic acid, and the solution was freeze-dried. To separate anthocyanins from other polyphenol compounds, the preparations were dissolved in 5% formic acid and processed using the ÄKTA Explorer 100 Air chromatograph (GE Healthcare, Chicago, IL, USA), equipped with a UV/VIS detector and an Agilent Zorbax SB C18 column (GE Healthcare). Two eluents were applied: 5% formic acid and methanol. The fraction of the effluent with an absorbance of 520 nm was collected and designated as the anthocyanin (ACN) fraction. Another fraction showing absorbance at 320 nm was collected and referred to as the non-anthocyanin polyphenol fraction (PP). Both fractions were evaporated, dissolved in an acetic acid-water solution, and freeze-dried. Both fractions were stored at −85 °C under a nitrogen atmosphere. The procedure for preparing the ACN and PP fractions has been described in detail in a previous work [17].

To analyze the bioactivity of RBF extract along with its RBF-ACN and RBF-PP fractions, freeze-dried samples were dissolved in a complete cell culture medium, and the pH was adjusted to 7.4. The resulting suspensions were centrifuged at 3000× *g* for 10 min., filtered through a 0.22 μm membrane, and then added to the cell cultures at concentrations ranging from 0.01 mg/mL to 1 mg/mL.

### 2.3. Polyphenol Identification and Quantification

RBF extract and RBF-ACN and RBF-PP fractions were analyzed using the HPLC-DAD-ESI-MS method on an Agilent 1200 series HPLC system (Agilent Technologies, Inc., Santa Clara, CA, USA), as previously described [17]. This system was equipped with a G1315D photodiode array detector and was coupled online with an Agilent 6224 time-of-flight MS system. Chromatographic separations were performed using a 150 × 2.1 mm, 3 μm C18 column (Advanced Chromatography Technologies, Aberdeen, Scotland). The mobile phase consisted of a mixture of 5% formic acid (phase A) and methanol (phase B). The separation was achieved using the following gradient: 5–25% B for 0–8 min, 25–45% B for 8–30 min, 45% B for 30–35 min, 45–5% B for 35–37 min, 5% B for 37–52 min. HPLC chromatograms were recorded at wavelengths of 280, 325, 355, and 520 nm, which are optimal for detecting flavan-3-ols, hydroxycinnamic acid derivatives, flavonols, and anthocyanins, respectively. The MS system, equipped with an electrospray ionization (ESI) source, was operated in both positive and negative ion modes, employing the following parameters: ion spray voltage of 4 kV (−2.9 kV), a capillary temperature of 325 °C, and nitrogen as the drying gas. A scan range of 100 to 1700 *m*/*z* enabled the identification of various phenolic compounds. Instrument control, data collection, and analysis were conducted using MassHunter B.04.00 software (Agilent Technologies, Inc.).

### 2.4. Cell Cultures and Anti-Inflammatory Experiment Procedure

Human umbilical vein endothelial cells (HUVEC; ATCC CRL-1730™) were sourced from the American Type Culture Collection (ATCC). HUVECs were cultured in F-12K medium (ATCC) supplemented with 10% FBS, an endothelial cell growth supplement derived from bovine neural tissue (30 µg/mL), and heparin (100 µg/mL). The cells were seeded at 6 × 10^3^ cells/cm^2^ onto 24-well plates coated with rat tail collagen. After 24 h, HUVECs were exposed to RBF extract at concentrations of 0.01, 0.1 and 1 mg/mL, as well as with RBF-ACN and RBF-PP fractions at concentrations of 0.1, 1 and 10 µg/mL for a duration of 3 h. Following this exposure, the cells were treated with TNF-α (10 ng/mL) for an additional 3 h to induce inflammation.

RAW 264.7 macrophages were obtained from the European Collection of Cell Cultures (ECACC, Cat. No 91062702) and cultured in DMEM supplemented with 10% FBS. For experiments, RAW 264.7 cells were seeded onto 24-well plates at an inoculum density of 5 × 10^5^ cells/cm^2^. After 24 h, the cells were treated with RBF extract at concentrations of 0.01, 0.1, and 1 mg/mL, as well as with RBF-ACN and RBF-PP fractions at concentrations of 0.1, 1, and 10 µg/mL for 1 h. Following this treatment, the cells were stimulated with LPS at a concentration of 1 μg/mL for an additional 3 h.

For each experiment, one positive control (cells treated with budesonide at 1 μM, a glucocorticoid steroid known for its potent anti-inflammatory properties) and one negative control (cells treated with a vehicle) were included.

HUVECs and RAW 264.7 cells were utilized for total RNA isolation and gene expression analysis. Additionally, the culture medium was collected to measure the secretion of IL-6 and MCP-1 proteins.

### 2.5. Cell Viability Assay

The viability of LPS-activated RAW 264.7 macrophages, both treated and untreated with RBF extract (at concentrations of 0.01, 0.1, and 1 mg/mL) as well as RBF-ACN and RBF-PP fractions (at concentrations of 0.1, 1, and 10 µg/mL), was analyzed using the MTT (3-(4,5-dimethylthiazol-2-yl)-2,5-diphenyltetrazolium bromide) assay following the procedure described previously [16]. Cells were seeded in 96-well plates at an initial density of 2.5 x 10^4^ cells/cm^2^. After 24 h of culture, the cells were treated with RBF extract and both fractions for either 24 or 48 h. Following treatment, the cells were exposed to MTT reagent (0.5 mg/mL) for 3 h. Insoluble formazan crystals were then extracted using isopropanol, and the absorbance was measured at 570 and 690 nm with a TECAN Infinite M200 microplate reader.

### 2.6. RNA Extraction and Real-Time PCR Analysis

RAW 264.7 macrophages and HUVECs were treated with TRI-Reagent for total RNA isolation. First-strand cDNA synthesis was performed using 1 µg of total RNA and a Transcriptor High Fidelity cDNA Synthesis Kit, following the manufacturer’s instructions. Gene expression quantification was carried out using a real-time PCR system (CFX96 Touch Real-Time PCR Detection System, Bio-Rad Laboratories, Inc., Hercules, CA, USA). The PCR mixture, with a final volume of 25 µL, included 1 µL of cDNA sample, 1 µL of specific forward and reverse primers (5 µM), and 12.5 µL of SYBR^®^ Select Master Mix. The primer sequences utilized in this experiment are provided in Table S1. PCR cycling conditions began with an initial denaturation at 94 °C for 10 min, followed by 40 PCR cycles: 40 s at 95 °C, 30 s at 59 °C, and 30 s at 72 °C. Relative gene expression was calculated using the 2^−ΔΔCT^ method. Transcript levels were normalized to β-actin for RAW 264.7 macrophages and to GAPDH for HUVECs. Relative mRNA expression was expressed as fold change compared with control (untreated) cells. All reactions were performed in triplicate.

### 2.7. Determination of IL-6 and MCP-1 Production

Protein concentrations of IL-6 and MCP-1 were measured using ELISA kits according to the manufacturer’s protocols (R&D Systems, Minneapolis, MN, USA). Quantitation was conducted by calibrating standards, and each standard and sample was assayed in triplicate.

### 2.8. Statistical Analysis

Statistical analysis was conducted using STATISTICA version 13.3 software (Statsoft, Inc., Tulsa, OK, USA). One-way analysis of variance (ANOVA) was employed, along with Tukey’s post hoc test, to evaluate the differences in mean values among multiple groups. Levene’s test was used to confirm the assumption of equal variances. A significance level of *p* ≤ 0.05 was applied to determine statistical significance.

## 3. Results

### 3.1. Composition of Polyphenols in Raspberry Fruit Extract and Its Anthocyanin and Non-Anthocyanin Fractions

The polyphenolic compounds found in freeze-dried RBF extract, as well as in RBF-ACN and RBF-PP fractions, are detailed in Table 1. In the RBF extract, the identified compounds were categorized as follows: anthocyanins (peaks 1–4), flavanols (peaks 5–7), hydroxycinnamic acid derivatives (peaks 8–11), and flavonols (peaks 12–19). The HPLC-DAD chromatograms for the extract and both fractions were obtained at the four wavelengths and are displayed in Figures S1–S3. The concentration of anthocyanins in the extract was determined to be 5.7 ± 0.39 mg/g. The predominant anthocyanins included cyanidin-3-*O*-sophoroside, cyanidin-3-*O*-glucoside, cyanidin-3-*O*-glucosyl-rutinoside, and cyanidin-3-*O*-rutinoside, which contributed an average of 53.9%, 24.7%, 12.5%, and 8.9% to the total anthocyanin content, respectively. Additionally, procyanidin B, catechin, and epicatechin were identified among the flavan-3-ols, with the total concentration of flavanols calculated at 1.00 ± 0.09 mg/g. In the RBF extract, free ellagic acid, its sugar conjugates, three quercetin, and one kaempferol glycoside were detected, although these were present in low concentrations. The most abundant quercetin glycoside was quercetin-3-*O*-galactoside, with an average concentration of 0.14 ± 0.01 mg/g (Table 1). The RBF-ACN fraction contained four main anthocyanin compounds: cyanidin-3-*O*-sophoroside (53.1%), cyanidin-3-*O*-glucosyl-rutinoside (13.0%), cyanidin-3-*O*-glucoside (24.0%), and cyanidin-3-*O*-rutinoside (9.9%) (Table 1).

The RBF-PP fraction contained polyphenolic compounds categorized into three groups: flavan-3-ols, hydroxycinnamic acid derivatives, and flavonols, which comprised 75.7%, 17.3%, and 7.0% of the total, respectively. Additionally, a residue of anthocyanin compounds (2.9%), including cyanidin-3-O-sophoroside, was detected in the RBF-PP fraction. The polyphenols present in significant amounts (greater than 5%) within the RBF-PP fraction included procyanidin B, catechin, epicatechin, caffeoyl hexoside, *p*-coumaryl hexoside isomer 1, and quercetin-3-*O*-galactoside (Table 1).

### 3.2. Anti-Inflammatory Effects of Raspberry Fruit Extract

**Figure 1:**
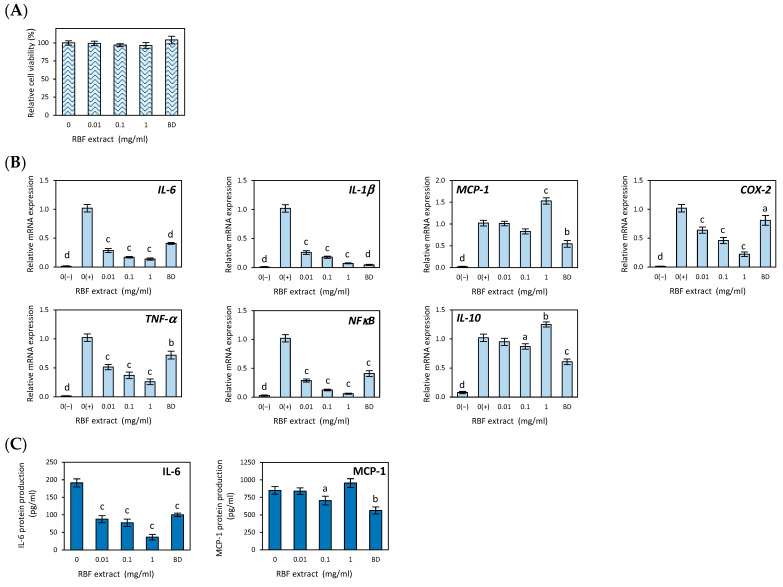
The effect of raspberry fruit extract (RBF) on the cell viability (**A**), inflammatory-related gene expression (**B**) and protein secretion (**C**) in LPS-stimulated RAW 264.7 macrophages. Budesonide (BD) was used as a positive control to verify anti-inflammatory effects. Data are the mean values ± SD (*n* = 3). Statistical analysis assessed differences between exposed cells and negative control 0(+) with significant differences indicated as follows: ^a^ *p* ≤ 0.05, ^b^ *p* ≤ 0.01, ^c^ *p* ≤ 0.001, ^d^ *p* ≤ 0.0001.

The cytotoxic effects of RBF extract on RAW 264.7 macrophages were evaluated using the MTT assay to identify non-cytotoxic doses for subsequent anti-inflammatory experiments. As shown in Figure 1A, RBF extract concentrations ranging from 0.01 to 1 mg/mL did not affect the viability of LPS-activated macrophages. This indicates that the observed anti-inflammatory effects were not due to cytotoxicity of the extract. In these experiments, macrophages were treated with LPS to stimulate an immune response and promote the release of inflammatory cytokines, as illustrated in Figure 1B.

The RBF extract and budesonide both reduced the increase in cytokine production induced by LPS in macrophages. At all tested non-cytotoxic doses, the RBF extract significantly inhibited the expression of the genes *IL-6*, *IL-1β*, *COX-2*, *TNF-α*, and *NF-κB* (Figure 1B). At the highest concentration of 1 mg/mL, the extract decreased the expression of these genes by 86%, 93%, 78%, 74%, and 99%, respectively (*p* ≤ 0.001). The inhibitory effects of the RBF extract at this concentration were more potent than those of budesonide (Figure 1B). Additionally, the RBF extract decreased the secretion of IL-6 protein in LPS-activated macrophages by 59% and 81% after treatment with 0.1 mg/mL and 1 mg/mL, respectively (*p* ≤ 0.001) (Figure 1C). In contrast, there was no statistically significant effect of the RBF extract on *MCP-1* gene expression (Figure 1B) or on MCP-1 protein secretion (Figure 1C). The extract also had a moderate impact on the mRNA level of IL-10, with a 25% increase in *IL-10* expression (*p* ≤ 0.01) observed only when LPS-induced macrophages were treated with the extract at the maximum dose of 1 mg/mL (Figure 1B).

### 3.3. Anti-Inflammatory Effects of Anthocyanin and Non-Anthocyanin Polyphenol Fractions of Raspberry Fruit Extract

**Figure 2:**
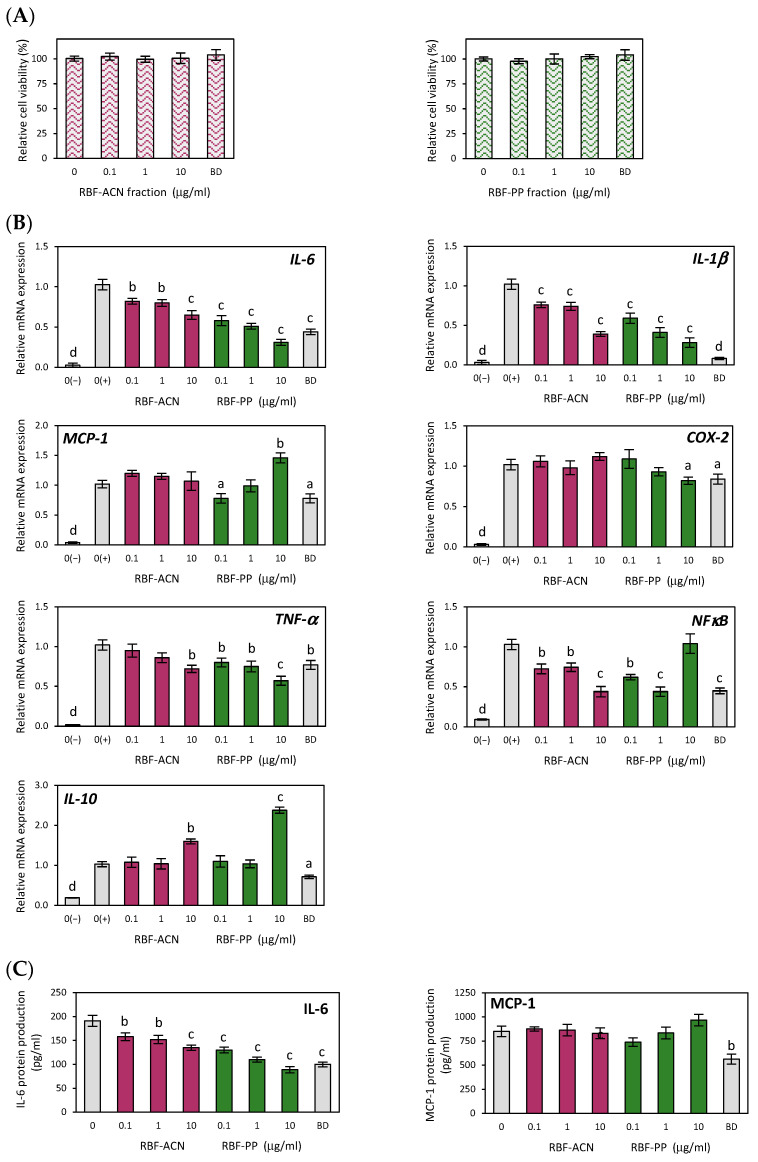
The effect of raspberry fruit anthocyanin fraction (RBF-ACN) and non-anthocyanin polyphenol fraction (RBF-PP) on the cell viability (**A**), inflammatory-related gene expression (**B**) and protein secretion (**C**) in LPS-stimulated RAW 264.7 macrophages. Budesonide (BD) was used as a positive control to verify anti-inflammatory effects. Data are the mean values ± SD (*n* = 3). Statistical analysis assessed differences between exposed cells and negative control 0(+) with significant differences indicated as follows: ^a^ *p* ≤ 0.05, ^b^ *p* ≤ 0.01, ^c^ *p* ≤ 0.001, ^d^ *p* ≤ 0.0001.

In this study, we investigated the anti-inflammatory potential of two fractions derived from RBF extract: the anthocyanin (RBF-ACN) and non-anthocyanin polyphenol (RBF-PP) fractions. First, we identified non-cytotoxic doses of the analyzed fractions suitable for anti-inflammatory experiments through the MTT test. As shown in Figure 2A, neither fraction affected macrophage viability at concentrations ranging from 0.1 µg/mL to 10 µg/mL. Real-time PCR analysis demonstrated significant suppressive effects of both RBF-PP and RBF-ACN fractions, evident by a dose-dependent decrease in the expression of pro-inflammatory markers, including IL-6, IL-1β, TNF-α, and NF-κB (Figure 2B). Notably, the RBF-PP fraction exhibited a slightly stronger effect. Depending on the dosage, the RBF-PP fraction reduced the expression of inflammatory mediators as follows: IL-6 (↓ 42–69%), IL-1β (↓ 41–72%), TNF-α (↓ 20–43%), and NF-κB (↓ 38–56%). The impact of the RBF-ACN fraction on IL-6 (↓18–35%), IL-1β (↓ 16–61%), and NF-κB (↓ 28–56%) expression was comparable to that of the RBF-PP fraction. A statistically significant effect on COX-2 expression was observed only when macrophages were treated with the RBF-PP fraction at a concentration of 10 µg/mL, resulting in an 18% decrease (*p* ≤ 0.05). This effect was similar to that achieved with budesonide. At the highest dose of 10 µg/mL, both fractions significantly upregulated IL-10 expression: the RBF-PP fraction increased IL-10 expression by 138% (*p* ≤ 0.001), while the RBF-ACN fraction resulted in a 60% increase (*p* ≤ 0.01) (Figure 2B). The secretion of the IL-6 cytokine in LPS-activated macrophage cultures changed in parallel with the altered expression levels induced by the RBF-ACN and RBF-PP fractions (Figure 2C). Similar to the whole RBF extract, neither the RBF-ACN nor the RBF-PP fractions had a statistically significant effect on MCP-1 gene expression and protein secretion (Figure 2B,C).

### 3.4. Potential of Raspberry Fruit Extract, Anthocyanin Fraction, and Non-Anthocyanin Polyphenol Fraction in Counteracting Vascular Endothelial Dysfunction

The inflammatory cytokine TNF-α plays a crucial role in impairing both macrovascular and microvascular circulation. Studies involving intra-arterial infusion of TNF-α in healthy volunteers provided direct evidence of TNF-α-induced vascular dysfunction. Research indicates that blocking or reducing the function of TNF-α may lower the risk of vascular complications associated with various diseases [18]. Markers of endothelial dysfunction include elevated plasma levels of soluble vascular cell adhesion molecule (sVCAM), soluble intercellular adhesion molecule (sICAM), endothelin-1, and E-selectin. Additionally, markers of low-grade inflammation, such as C-reactive protein (CRP), TNF-α, IL-6, and IL-1β, are also indicative of this dysfunction [18].

**Figure 3:**
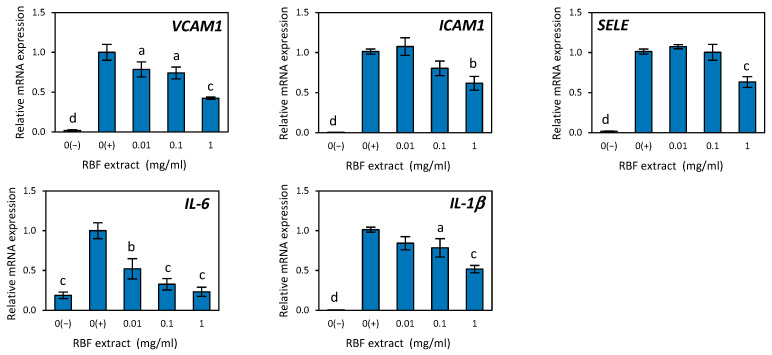
The effect of raspberry fruit (RBF) extract on the expression of inflammatory-related genes in HUVECs treated with TNF-α. The negative control 0(−) was not treated with TNF-α. Data are the mean values ± SD (*n* = 3). Statistical analysis assessed differences compared to the positive control 0(+), with significant differences indicated as follows: ^a^ *p* ≤ 0.05, ^b^ *p* ≤ 0.01, ^c^ *p* ≤ 0.001, ^d^ *p* ≤ 0.0001.

**Figure 4:**
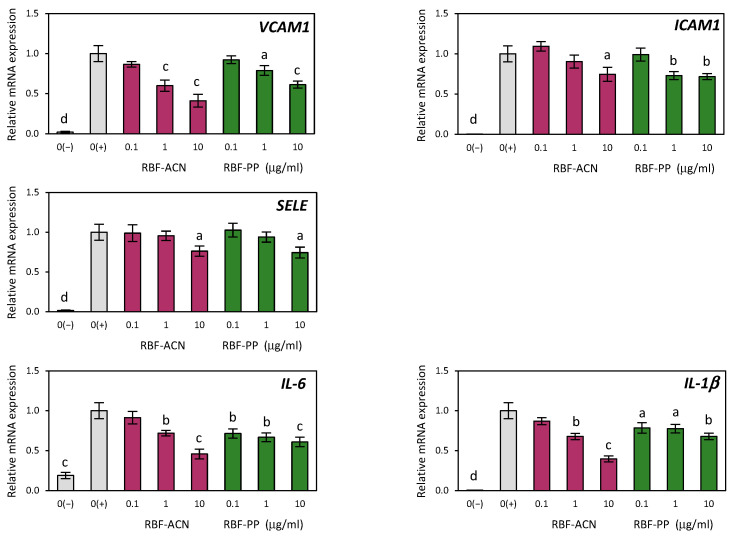
The effects of anthocyanin (RBF-ACN) and non-anthocyanin polyphenol (RBF-PP) fractions on the expression of inflammatory-related genes in HUVECs treated with TNF-α. The negative control 0(−) was not induced by TNF-α. Data are the mean values ± SD (*n* = 3). Statistical analysis assessed differences compared to the positive control 0(+) with significant differences indicated as follows: ^a^ *p* ≤ 0.05, ^b^ *p* ≤ 0.01, ^c^ *p* ≤ 0.001, ^d^ *p* ≤ 0.0001.

In this study, we examined the effectiveness of RBF extract, as well as the RBF-ACN and RBF-PP fractions, in counteracting endothelial inflammation induced by TNF-α in HUVECs. TNF-α significantly increased the expression of various inflammation-related cytokines and adhesion molecules in HUVECs, including IL-6, IL-1β, VCAM-1, ICAM-1, and SELE (Figure 3 and Figure 4). Compared to the control cells, the RBF extract, along with the RBF-PP and RBF-ACN fractions, decreased the TNF-α-induced increase in the expression of IL-6, IL-1β, VCAM-1, ICAM-1, and SELE in a dose-dependent manner (Figure 3 and Figure 4).

After incubating HUVECs with RBF extract at concentrations of 0.1 µg/mL and 1 µg/mL, we observed a statistically significant reduction in the mRNA expression levels of *IL-6* and *IL-1β*. Specifically, *IL-6* mRNA expression decreased by 67% and 77% (*p* ≤ 0.001), while *IL-1β* expression decreased by 22% (*p* ≤ 0.05) and 48% (*p* ≤ 0.001). Additionally, the effects of RBF extract on the expression of *VCAM-1*, *ICAM-1*, and *SELE* were significant at a concentration of 1 mg/mL. *VCAM-1* expression decreased by 58% (*p* ≤ 0.001), *ICAM-1* by 39% *(p* ≤ 0.001), and *SELE* by 37% (*p* ≤ 0.001) (Figure 3).

The RBF-ACN and RBF-PP fractions exhibited the strongest inhibitory effects on the mRNA expression of *IL-6*, *IL-1β*, *VCAM-1*, *ICAM-1*, and *SELE* at doses of 1 µg/mL and 10 µg/mL. Notably, the RBF-ACN fraction had a slightly more pronounced impact compared to the RBF-PP fraction. Specifically, the RBF-ACN fraction reduced *IL-6* expression by 28% and 54%, *IL-1β* by 32% and 60%, and *VCAM-1* by 40% and 60%. The RBF-PP fraction decreased *IL-6* transcripts by 33% and 39%, *IL-1β* by 23% and 32%, and *VCAM-1* by 21% and 39% (Figure 4). Significant reductions in *ICAM-1* and *SELE* expression were observed only at the higher dose of 10 µg/mL for both fractions. The RBF-ACN and RBF-PP fractions similarly lowered the mRNA expression of *ICAM-1* and *SELE*, with the RBF-ACN fraction decreasing *ICAM-1* expression by 25% and 28% and *SELE* expression by 24% and 25%, respectively (Figure 4).

## 4. Discussion

Chronic inflammation plays a significant role in the development and progression of various disorders, including type 2 diabetes mellitus, CVD, atherosclerosis, and neurodegenerative diseases. Therefore, targeting inflammation presents a promising strategy for addressing inflammation-related conditions [19]. Abnormalities in lipid metabolism, endothelial dysfunction, and the infiltration of inflammatory cells are key contributors to the development of atherosclerosis. Macrophages are vital in both the progression and regression of atherosclerotic disease [1]. They release pro-inflammatory cytokines, such as IL-1β, IL-6, and TNF-α, in response to various inflammatory stimuli. However, macrophages also play an essential role in stabilizing and regressing plaques [1]. Plaque macrophages facilitate tissue repair and angiogenesis by releasing anti-inflammatory cytokines like IL-10 and TGF-β [1,5]. Reducing the inflammatory response of macrophages has been linked to anti-atherogenic effects; many potential treatments for CVD aim to target the pro-inflammatory cytokines released by these cells. In vivo studies have shown that inhibiting IL-1β expression in macrophages significantly reduces cardiovascular events [20].

Evidence from prospective studies and randomized controlled trials suggests that more than half of all cardiovascular events could be prevented by improving diet [10]. Research, both in vivo and in vitro, indicates that polyphenols found in berry fruits may help reduce inflammation. Additionally, consuming foods rich in anthocyanins is associated with a lower risk of chronic, non-communicable diseases [19]. Dietary anthocyanins and other phenolic compounds may influence inflammation by modulating the expression of pro-inflammatory and anti-inflammatory cytokines [21]. Common sources of anthocyanins include blueberries, blackberries, elderberries, aronia fruit, and raspberries [19].

This study assessed the anti-inflammatory effects of the whole RBF extract and its fractions, RBF-ACN and RBF-PP, by measuring their impact on pro-inflammatory and anti-inflammatory gene expression in LPS-activated RAW 264.7 macrophages. The results demonstrated that the mRNA expression levels of IL-6, IL-1β, COX-2, TNF-α, and NF-κB were significantly reduced by the whole RBF extract, which showed a stronger effect than budesonide. While both fractions, RBF-ACN and RBF-PP, also inhibited the expression of pro-inflammatory cytokines, their impact was not as pronounced as that of the whole extract. These findings suggest that interactions among multiple components could significantly enhance the anti-inflammatory potential. Additionally, at the highest doses, the RBF extract, RBF-ACN fraction, and RBF-PP fraction increased the mRNA expression of IL-10 in LPS-activated macrophages.

Several previous studies have reported that anthocyanins found in berries can modulate inflammatory responses in cell culture and animal models. For example, Li et al. investigated the anti-inflammatory effects of crude extracts, anthocyanin-rich fractions, and des-anthocyanin fractions from seven berries, including red raspberry, black raspberry and mulberry, using LPS-activated RAW 264.7 macrophages. Their research revealed that the anthocyanin fractions derived from red raspberries were the most effective at inhibiting the expression of iNOS and COX-2. Furthermore, raspberry anthocyanin fractions significantly reduced the mRNA levels of IL-1β and IL-6. These fractions also suppressed the NF-κB and AP-1 signaling pathways in RAW 264.7 cells in a concentration-dependent manner [22].

Previous research has shown that the anthocyanin fraction from blueberries, blackberries, and blackcurrants at 10 µg/mL or 20 µg/mL exhibits significant anti-inflammatory effects in LPS-activated macrophages. These effects result from the downregulation of IL-1β and TNF-α expression, primarily through the inhibition of the NF-κB pathway [23]. Studies indicate that treatment of LPS-stimulated human THP-1 macrophages with cyanidin-3-*O*-β-glucoside significantly reduces the expression and secretion of pro-inflammatory cytokines, such as TNF-α and IL-6 [24]. Furthermore, a dietary intervention using red raspberry fruit (5.3% freeze-dried raspberry) in obese diabetic mice over 8-week period led to a decrease in IL-6 plasma concentration [25]. In a study by Mykkanen et al., the incorporation of Nordic wild blueberries (10% by weight) into a high-fat diet (comprising 45% fat) for 12 weeks effectively reduced serum concentrations of key pro-inflammatory markers, including IL-1, IL-2, and TNF-α, in C57BL/6 mice [26].

In a randomized controlled trial, daily supplementation with 250 g of frozen red raspberry for 4 weeks was associated with significantly lower levels of inflammation biomarkers, particularly IL-6 and TNF-α, in adults with established type 2 diabetes mellitus [27]. Additionally, in individuals with metabolic syndrome, 4 weeks of consuming anthocyanin-rich berries significantly down-regulated the expression of NF-κB-dependent genes, including *TNF-α*, *IL-6*, *IL-1A*, *PCAM-1*, and *COX-2*. These results suggest that berry supplementation may help alleviate features of metabolic syndrome and related cardiovascular risk factors, potentially due to the inhibition of NF-κB-dependent gene expression [28]. NF-κB is a key regulator of various genes involved in inflammation. The activation of NF-κB by kinases promotes the transcription of genes that encode pro-inflammatory chemokines and cytokines. Consequently, reducing NF-κB activation is being investigated as a potential strategy for preventing chronic inflammatory diseases [29]. Numerous studies have shown that downregulating NF-κB signaling is the primary mechanism through which berries and their bioactive compounds exert their anti-inflammatory effects [28,29,30]. Our research showed that the RBF extract, along with the RBF-ACN and RBF-PP fractions, significantly suppressed the expression of NF-κB induced by LPS. This finding suggests that the anti-inflammatory effects of raspberry phytochemicals are achieved by inhibiting the activation of the NF-κB pathway.

Inflammation plays a crucial role in causing dysfunction of the vascular endothelial by activating transcription factors such as NF-κB. The activation of the NF-κB leads to increased expression of pro-inflammatory cytokines (TNF-α, IL-1β, IL-6), chemokines, and adhesion molecules (ICAMs and VCAM-1) [24]. Pro-inflammatory cytokines related to vascular macrophages trigger inflammation-mediated increases in vascular permeability [31]. Our data demonstrated that RBF extract, along with the RBF-ACN and RBF-PP fractions, has a protective effect on endothelial functions by reducing the expression of several inflammation-related genes and adhesion molecules, including IL-6, IL-1β, VCAM-1, ICAM-1, and SELE in TNF-α-induced HUVECs. In vitro studies and in vivo animal models have evaluated the effects of raspberry polyphenolic components or raspberry fruit extracts, showing their efficacy in improving endothelial function [11].

In cell culture studies, a purified anthocyanin mixture was found to inhibit CRP induced by IL-6 and IL-1β in the HepG2 cell line. It also reduced the secretion of VCAM-1 triggered by LPS in the porcine iliac artery endothelial cell line. Notably, the anti-inflammatory effects of the anthocyanin mixture were more significant than those of delphinidin-3-*O*-β-glucoside and cyanidin-3-*O*-β-glucoside when administered separately, suggesting that the components in the anthocyanin mixture may work additively or synergistically to enhance anti-inflammatory responses [32]. Furthermore, when HUVECs were pretreated with cyanidin-3-*O*-glucoside, there was a significant reduction in TNF-α-induced expression of adhesion molecules such as ICAM-1, VCAM-1, and E-selectin, along with an inhibition of NF-κB activation [33].

In studies with sea buckthorn berry extract, the polyphenols significantly reduced serum levels of TNF-α and IL-6 and also alleviated vascular impairment by decreasing the expression of eNOS and ICAM-1 in the aortas of rats with hyperlipidemia [34]. Additionally, female patients with metabolic syndrome, who consumed 4 cups of freeze-dried strawberries daily for 8 weeks experienced lower levels of LDL cholesterol and VCAM-1 [35]. In adults with hypercholesterolemia, a purified anthocyanin mixture led to significant reductions in serum CRP, sVCAM-1, and plasma IL-1β compared to a placebo [32]. Finally, in patients with metabolic syndrome who consumed black raspberry for 12 saw significant decreases in total serum cholesterol levels and inflammatory cytokines, including IL-6, TNF-α, CRP, sICAM-1, and sVCAM-1, ultimately improving vascular endothelial function [36].

The bioavailability of the bioactive components found in red raspberries and other berries is influenced by several factors. These include the food matrix, the amount consumed, the timing of consumption, and interactions with gut microbiota. Research suggests that the health benefits of dietary berries can be effectively achieved through daily intake of 40–250 g of fresh, frozen, or dried berries, or their extracts [37].

In summary, the results have shown that RBF extract and its two analyzed fractions exhibit a strong anti-inflammatory effect in macrophage cell cultures. This effect is achieved by inhibiting the LPS-induced expression of pro-inflammatory genes such as IL-6, IL-1β, TNF-α, and NF-κB highlighting the protective role of raspberries in preventing chronic inflammation. Additionally, the RBF extract, along with the RBF-ACN and RBF-PP fractions, demonstrated a protective effect on endothelial function. This was evidenced by a decrease in the expression of various inflammation-related genes and adhesion molecules, including IL-6, IL-1b, VCAM-1, ICAM-1, and SELE in TNF-α-induced HUVECs. These findings suggest that consuming raspberry fruit may help prevent the early development of atherosclerosis, with mechanisms linked to improved inflammation status and vascular endothelial function. Overall, the data indicate that raspberry fruit supplementation could play a role in the prevention or treatment of chronic inflammatory diseases by inhibiting pro-inflammatory chemokines, cytokines, and inflammatory mediators. Therefore, raspberries may serve as an effective dietary agent for addressing inflammatory and cardiovascular complications.

## 5. Conclusions

Chronic inflammation is a key factor in the development and progression of various disorders, including atherosclerosis, autoimmune diseases, neurodegenerative diseases, and cardiovascular complications. Therefore, creating effective anti-inflammatory drugs poses a significant challenge. Diet is a crucial element that can lower the risk of inflammation-related diseases by the ability of certain foods and their bioactive compounds to reverse or prevent the progression of the pathogenic inflammatory processes that underlie these diseases. Our studies provide evidence that incorporating raspberry fruits, known for their significant anti-inflammatory effects, into the daily diet can significantly reduce the risk of non-communicable diseases related to inflammation.

Future research trials involving human participants are needed to confirm the health benefits of long-term supplementation with berry fruits. These studies will help determine whether red raspberries and other berries can prevent and treat disorders. Additionally, they will investigate the effectiveness of dietary interventions before pharmaceutical intervention. It is also important to clarify the dose-dependent effects of red raspberry supplementation, including the safety and effectiveness of different dosages, and to identify the optimal therapeutic dose.

## Figures and Tables

**Table 1:** Identification and quantification of phenolic compounds contained in raspberry fruit (RBF), RBF anthocyanin (RBF-ACN), and RBF non-anthocyanin (RBF-PP) extracts. Quantitative data are expressed as mg/g lyophilized powder.

Peak No.	RT (min)	UV λ_max_ (nm)	[M − H]−/ /[M + H]+ (*m*/*z*)	MS/MS (*m*/*z*)	Tentative Identification	Concentration (mg/g) *		
						RBF	RBF-ACN	RBF-PP
RBF anthocyanins								
1	15.77	280, 520	611.1642 (+)	449.1078	Cyanidin-3-*O*-sophoroside	3.07 ± 0.18	520.02 ± 12.54	28.29 ± 1.95
				287.0615				
2	16.44	280, 520	757.2220 (+)	595.1663	Cyanidin-3-*O*-glucosyl-rutinoside	0.71 ± 0.05	127.25 ± 6.23	Trace amounts
				287.0572				
3	17.18	280, 520	449.1123 (+)	287.0589	Cyanidin-3-*O*-glucoside	1.41 ± 0.12	235.26 ± 5.22	Trace amounts
4	18.20	280, 520	595.1734 (+)	287.0578	Cyanidin-3-*O*-rutinoside	0.51 ± 0.04	97.25 ± 3.41	Trace amounts
RBF flavanols								
5	9.84	280	577.1355 (−)	289.0712	Procyanidin B	0.30 ± 0.03	-	42.43 ± 2.05
6	10.74	280	289.1139 (−)	245.1203	Catechin	0.12 ± 0.02	-	26.06 ± 1.13
7	12.61	280	289.1142 (−)	245.1210	Epicatechin	0.58 ± 0.04	-	140.39 ± 5.97
RBF hydroxycinnamic acid derivatives								
8	9.61	278, 320	341.0880 (−)	161.0238	Caffeoyl hexoside	0.10 ± 0.01	-	19.98 ± 0.96
9	12.60	234, 316	325.0933 (−)	145.0294	*p*-Coumaryl hexoside isomer 1	0.08 ± 0.01	-	27.72 ± 1.08
10	14.30	234, 316	325.0933 (−)	145.0293	*p*-Coumaryl hexoside isomer 2	0.07 ± 0.01	Trace amounts	Trace amounts
11	14.93	235, 325	163.0402 (−)	119.0496	*p*-Coumaric acid	0.09 ± 0.02	Trace amounts	Trace amounts
RBF flavonols								
12	22.19	254, 360	433.0417 (−)	300.9982	Ellagic acid-*O*-pentoside	Trace amounts	-	Trace amounts
13	23.35	262, 363	300.9992 (−)	229.0131	Ellagic acid	0.21 ± 0.03	-	Trace amounts
14	23.69	266, 354	463.0874 (−)	301.0334	Quercetin-3-*O*-galactoside	0.14 ± 0.01	-	19.45 ± 1.37
15	24.41	268, 354	463.0882 (−)	301.0354	Quercetin-3-*O*-glucoside	Trace amounts	-	Trace amounts
16	24.62	272, 355	477.0679 (−)	301.0345	Quercetin 3-*O*-glucuronide	Trace amounts	-	Trace amounts
17	25.88	266, 348	461.0733 (−)	285.0391	Kaempferol 3-*O*-glucuronide	Trace amounts	-	Trace amounts
18	28.45	256, 360	475.0525 (−)	300.9978	Ellagic acid acetyl pentoside isomer 1	Trace amounts	-	Trace amounts
19	29.52	256, 360	475.0525 (−)	299.9904	Ellagic acid acetyl pentoside isomer 2	Trace amounts	-	Trace amounts

* Values were expressed as mean ± SEM for three independent experiments.

## Data Availability

The raw data supporting the conclusions of this article will be made available by the authors on request due privacy reason.

## Supplementary Materials

The following supporting information can be downloaded at https://www.mdpi.com/article/10.3390/nu17203257/s1: Figure S1: HPLC-DAD chromatograms of raspberry fruit (RBF) extract recorded at 280 (A), 520 (B), 325 (C), and 355 nm (D) for optimal detection of flavan-3-ols, anthocyanins, hydroxycinnamic acid derivatives, and flavonols, respectively (peak numbers correspond to those in Table 1); Figure S2: HPLC-DAD chromatograms of anthocyanin raspberry fruit (RBF-ACN) extract recorded at 280 (A), 520 (B), 325 (C), and 355 nm (D) for optimal detection of flavan-3-ols, anthocyanins, hydroxycinnamic acid derivatives, and flavonols, respectively (peak numbers correspond to those in Table 1); Figure S3: HPLC-DAD chromatograms of raspberry fruit polyphenol (RBF-PP) extract recorded at 280 (A), 520 (B), 325 (C), and 355 nm (D) for optimal detection of flavan-3-ols, anthocyanins, hydroxycinnamic acid derivatives, and flavonols, respectively (peak numbers correspond to those in Table 1); Table S1: The primers sequence used for real-time PCR.
