# Supplementary material for: Immunomodulatory Effect of Raspberry (Rubus idaeus L.) Fruit Extracts on Activated Macrophages and Dysfunctional Vascular Endothelial Cells
Source: Nutrients. 2025 Oct 16;17(20):3257. doi: 10.3390/nu17203257 (PMC12566686; doi:10.3390/nu17203257)
Supplement: Supplementary file 1 [file nutrients-17-03257-s001.zip › nutrients-3905608-supplementary.pdf]

Table S1. The primers sequence used for real-time PCR

Gene	Accession	No.Sequence (5′ – 3′)	Amplicon (bp)
**Mm *IL-6***	NM-031168.1	F:TCTGAAGGACTCTGGCTTTG<br>R:GATGGATGCTACCAAACTGGA	142
**Mm *IL-1β***	NM-008361.3	F:AGGTCAAAGGTTTGGAAGCA<br>R:TGAAGCAGCTATGGCAACTG	129
**Mm *IL-10***	NM-010548.2	F:CAGGGATCTTAGCTAACGGAAAC<br>R:GCTCAGTGAATAAATAGAATGGGAAC	110
**Mm *NF-κβ***	NM-019408.3	F:CTGGTGGACACATACAGGAAGAC<br>R:ATAGGCACTGTCTTCTTTCACCTC	195
**Mm** *TNF-α*	NM-001278601.1	F:TCTACTGAACTTCGGGGTGA<br>R:AGGGTCTGGGCCATAGAACT	87
**Mm *MCP-1***	NM-011333.3	F:TTCCTCCACCACCATGCAG<br>R:CCAGCCGGCAACTGTGA	64
**Mm** *COX-2*	NM-011198.3	F:GGCGCAGTTTATGTTGTCTGT<br>R:CAAGACAGATCATAAGCGAGGA	107
**Mm *ACTB***	NM-007393	F:CCA CAG CTG AGA GGG AAA TC<br>R:AAG GAA GGC TGG AAA AGA GC	193
**Hs *GAPDH***	NM-008084	F: AATGAAGGGGTCGTTGATGG<br>R: ATGGTGAAGGTCGGTGTGAA	108
**Hs** *IL-6*	NM-001371096.1	F:GCAGAAAACAACCTGAACCTT<br>R:ACCTCAAACTCCAAAAGACCA	116
**Hs *VCAM-1***	NM-001078.4	F:CAGGCTAAGTTACATATTGATGACAT<br>R:GAGGAAGGGCTGACCAAGAC	116
**Hs *ICAM-1***	NM-000201.3	F:GTATGAACTGAGCAATGTGCAAG<br>R:GTTCCACCCGTTCTGGAGTC	119
**Hs** *SELE*	NM-000450.2	F: AGAGTGGAGCCTGGTCTTACA<br>R: CCTTTGCTGACAATAAGCACTGG	77
**Hs** *IL-1β*	NM-000576.3	F:CCTGAAGCCCTTGCTGTAGT<br>R:AGCTGATGGCCCTAAACAGA	112

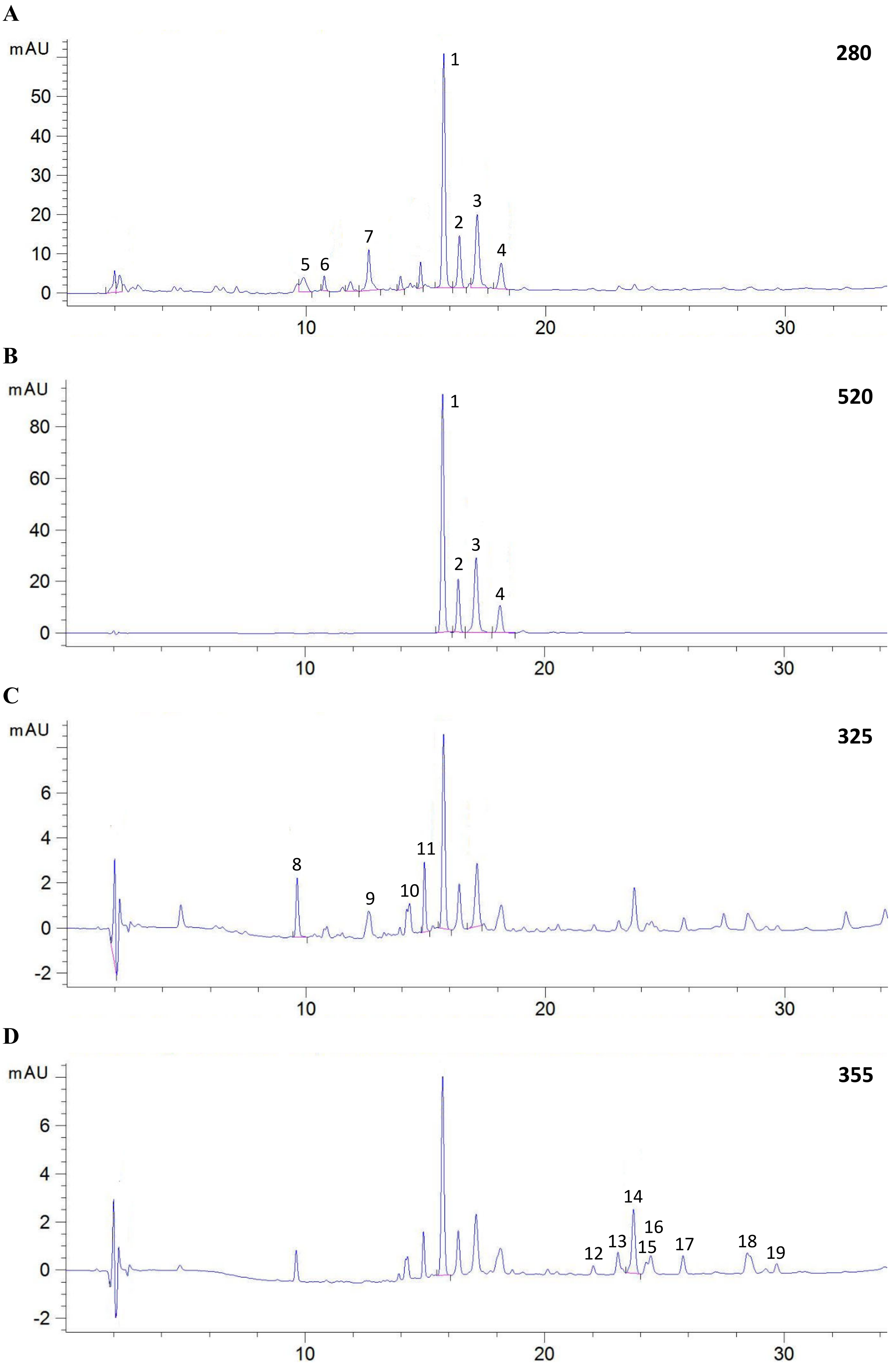

A
mAU
280
0
10
20
30
40
50
1
2
3
4
5
6
7
10
20
30
B
mAU
520
0
20
40
60
80
1
2
3
4
10
20
30
C
mAU
325
-2
0
2
4
6
8
9
10
11
10
20
30
D
mAU
355
-2
0
2
4
6
12
13
14
15
16
17
18
19
10
20
30

Figure S1. HPLC-DAD chromatograms of raspberry fruit (RBF) extract recorded at 280 (A), 520 (B), 325 (C), and 355 nm (D) for optimal detection of flavan-3-ols, anthocyanins, hydroxycinnamic acid derivatives, and flavonols, respectively (peak numbers correspond to those in Table 1).

A

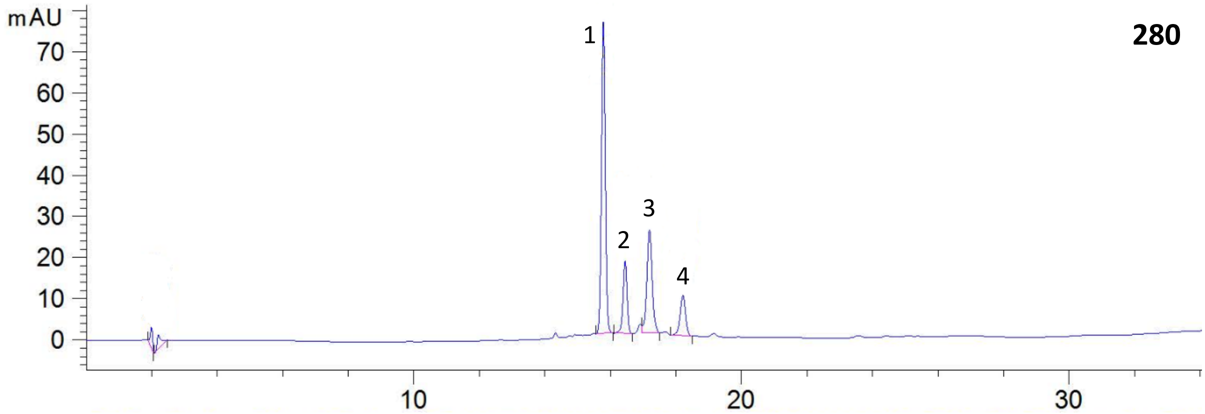

B

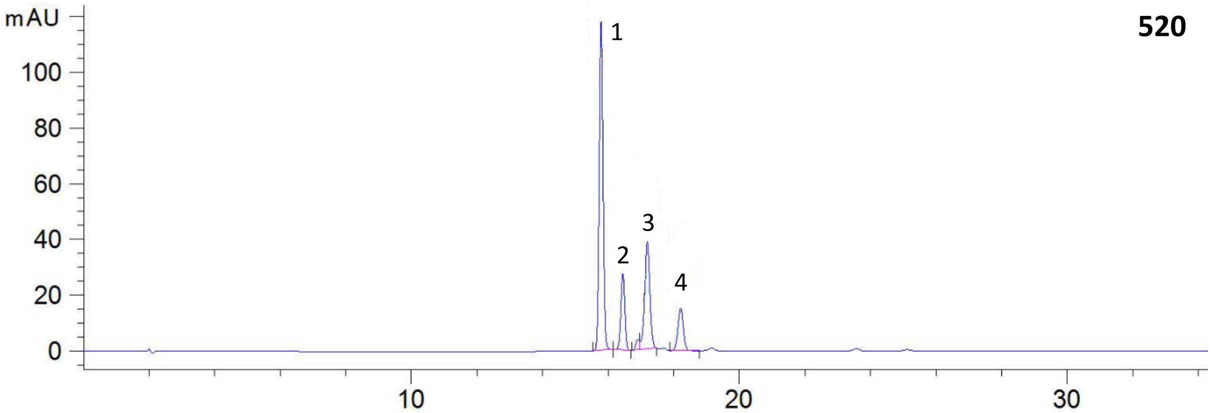

C

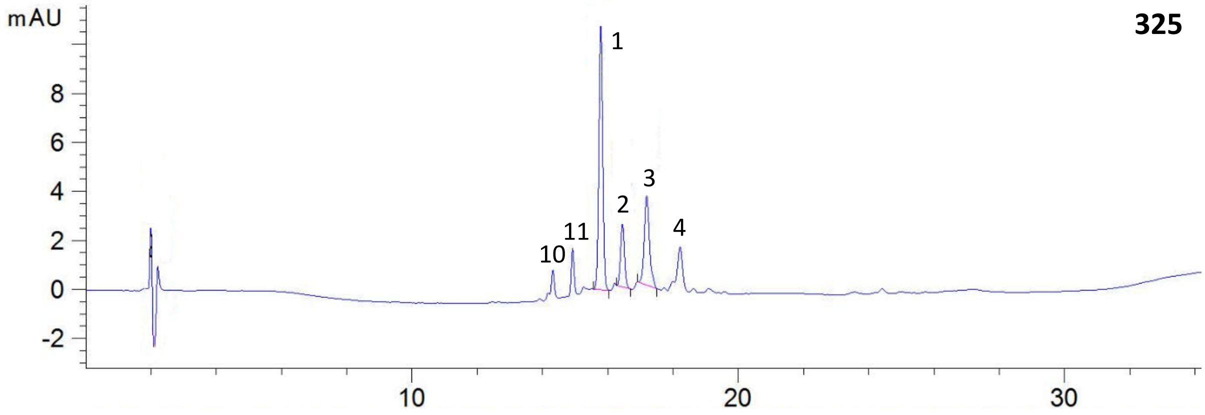

D

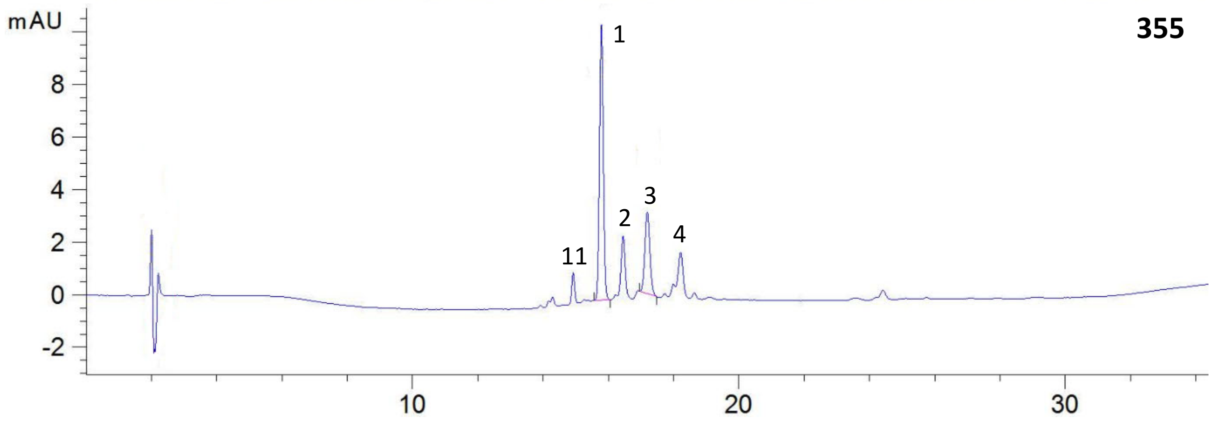

Figure S2. HPLC-DAD chromatograms of anthocyanin raspberry fruit (RBF-ACN) extract recorded at 280 (A), 520 (B), 325 (C), and 355 nm (D) for optimal detection of flavan-3-ols, anthocyanins, hydroxycinnamic acid derivatives, and flavonols, respectively (peak numbers correspond to those in Table 1).

A

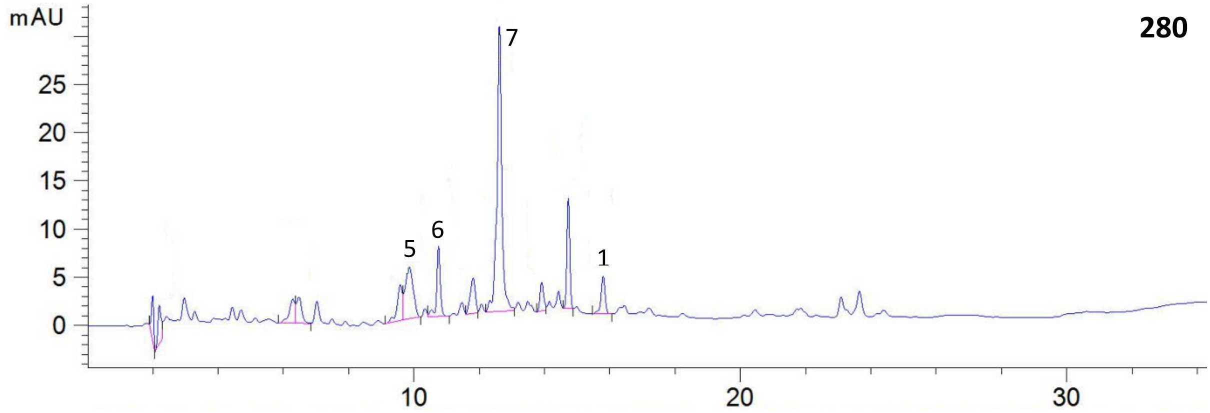

B

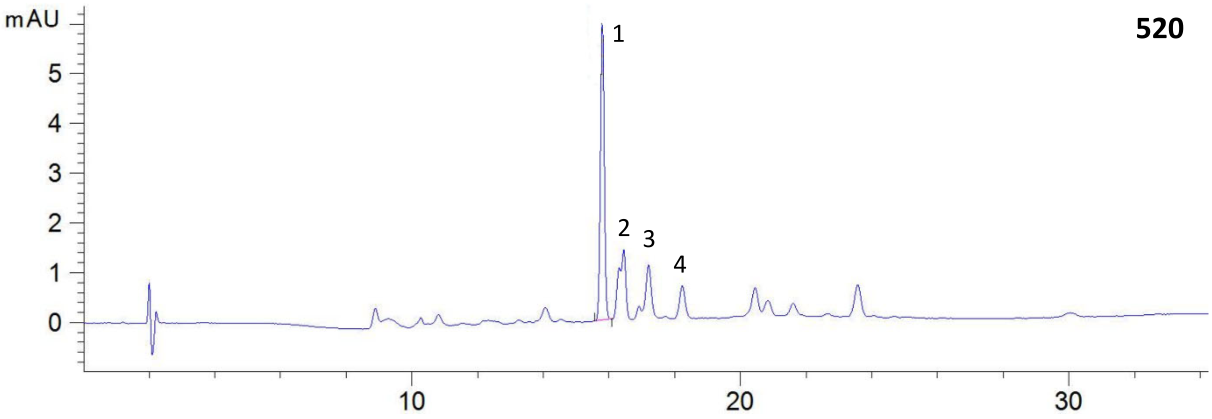

C

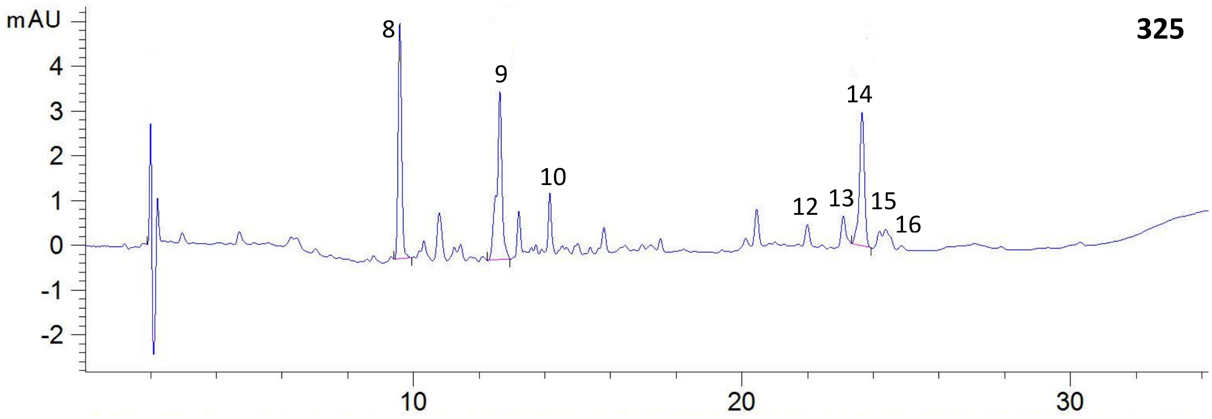

D

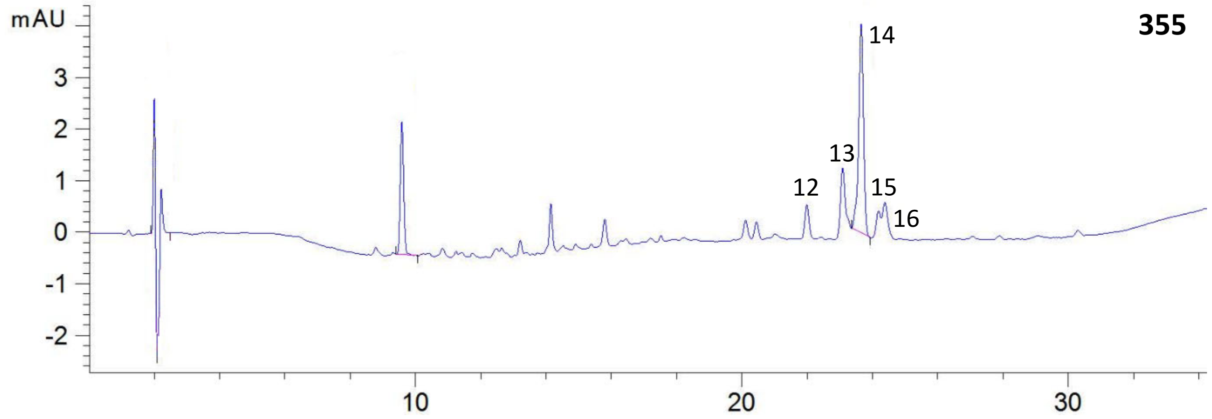

Figure S3. HPLC-DAD chromatograms of raspberry fruit polyphenol (RBF-PP) extract recorded at 280 (A), 520 (B), 325 (C), and 355 nm (D) for optimal detection of flavan-3-ols, anthocyanins, hydroxycinnamic acid derivatives, and flavonols, respectively (peak numbers correspond to those in Table 1).
